# Supplementary material for: Graphasing: phasing diploid genome assembly graphs with single-cell strand sequencing
Source: Genome Biol. 2024 Oct 10;25:265. doi: 10.1186/s13059-024-03409-1 (PMC11466045; doi:10.1186/s13059-024-03409-1)
Supplement: Supplementary file 1 — Supplementary Material 1: Fig. S1. Strand-seq Phasing Signal. Fig. S2. Hairpin-Capped Broken Bubbles Interrupt Rukki Scaffolding. Fig. S3. Sex Chromosome Hap-mer Blob Plots. Fig. S4. Behavior of Absolute SSF Calculated with bwa mem Alignments. Fig. S5. Haplotype, Cluster, and Phase Vectors. Fig. S6. Read Labeling for Proper Strand-seq Library Pooling [file 13059_2024_3409_MOESM1_ESM.docx]

# Additional File 1

## **Supplementary Figures**

### for

## Graphasing: phasing diploid genome assembly graphs with single-cell strand sequencing

Mir Henglin, Maryam Ghareghani, William Harvey, David Porubsky, Sergey Koren, Evan E. Eichler, Peter Ebert, Tobias Marschall

Table of Contents:

- Fig. S1. Strand-seq Phasing Signal
- Fig. S2. Hairpin-Capped Broken Bubbles interrupt Rukki Scaffolding.
- Fig. S3. Sex Chromosome Hap-mer Blob Plots.
- Fig. S4. Behavior of Absolute SSF Calculated with bwa mem Alignments.
- Fig. S5. Haplotype, Cluster, and Phase Vectors.
- Fig. S6. Read Labeling for Proper Strand-seq Library Pooling


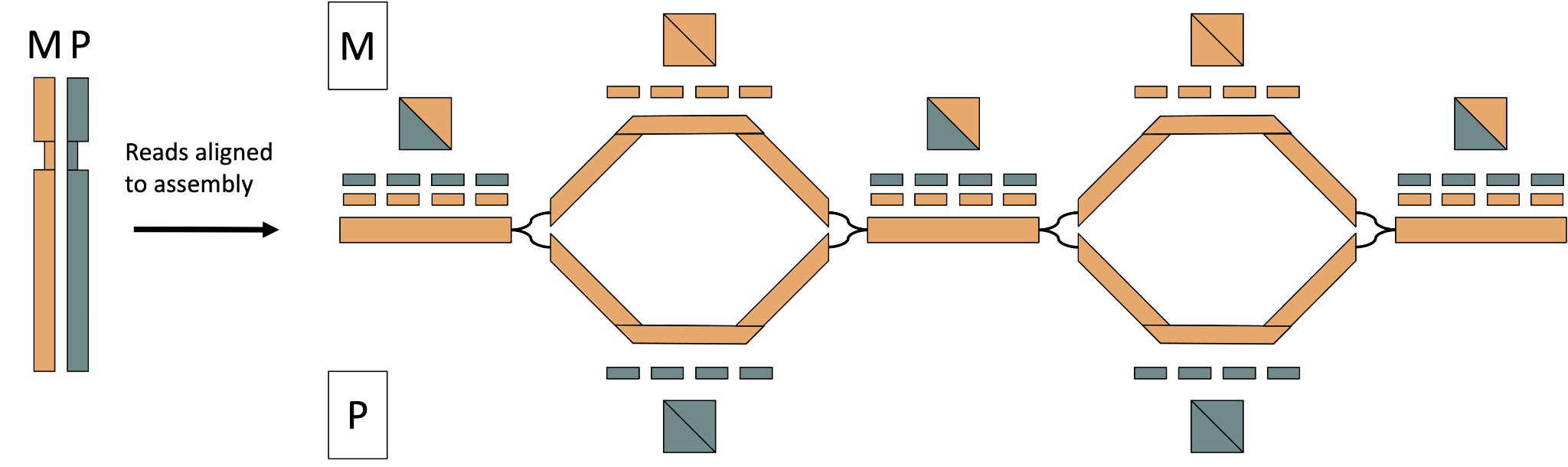


*Fig. S1. Strand-seq Phasing Signal. A reads from a WC Strand-seq library (left) are aligned to the unitigs of a chromosome (right). Orange shapes correspond to Watson orientation, and green shapes correspond to Crick orientation. The graph unitigs and maternal reads are all in Watson orientation, while the paternal reads are all in Crick orientation. Consequently, sequence unique to the maternal haplotype will receive only “forward” alignments (orange box), while sequence unique to the paternal haplotype will receive only “reverse” alignments (green box). Sequence shared between the haplotypes receives a 50/50 mix of forward and reverse alignments (green\orange box)*


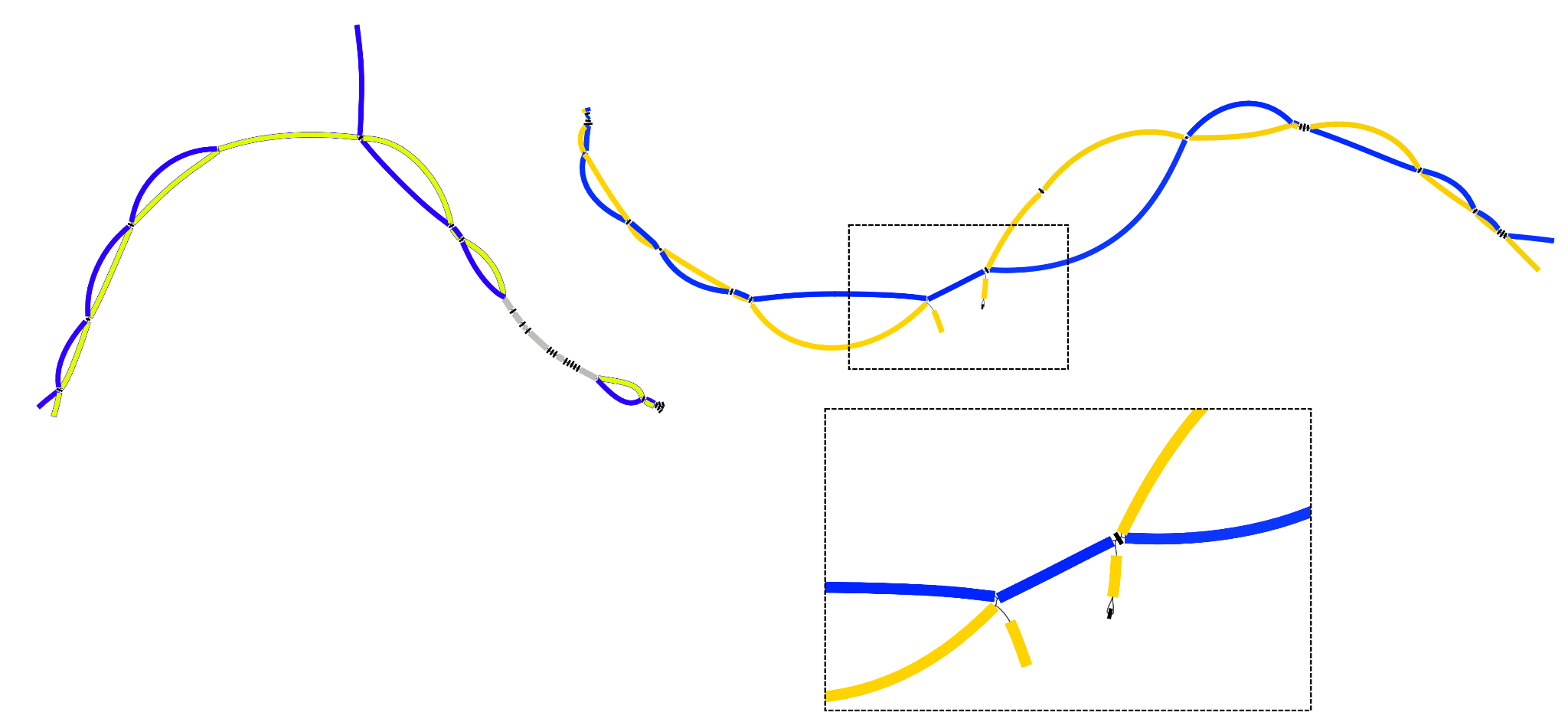


*Fig. S2. Hairpin-Capped Broken Bubbles Interrupt Rukki Scaffolding. Bandage [67] graphs of chromosomes 5 (left) and 1 (right, top) from the Verkko HG00733 assembly both show broken bubbles. On each connected component, the blue or yellow shade corresponds to a haplotype as inferred by Graphasing, grey corresponds to homozygous nodes, and black to nodes without inferred phasing signal. The blue haplotype on chromosome 5 is scaffolded in one path, but the yellow haplotype of chromosome 1 is represented with two scaffolds, one on either side of the broken bubble, and is the only pictured haplotype not connected in a single path. Zooming in (right, bottom) shows that one end of the broken bubble is “capped” by a single small node in a hairpin structure.*


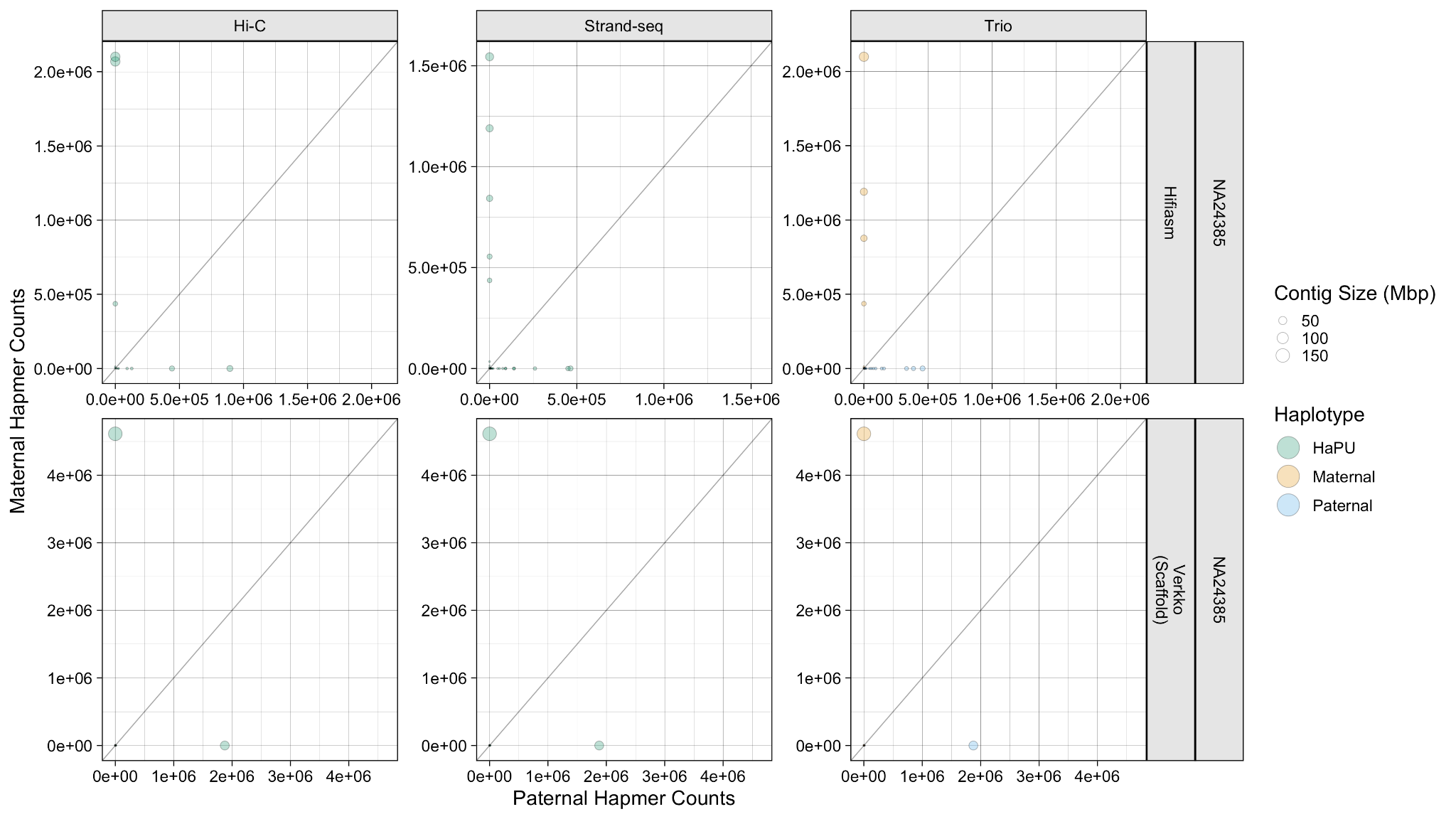


*Fig. S3. Sex Chromosome Hap-mer Blob Plots. NA24385 contigs aligning to the X and Y chromosomes are plotted.*


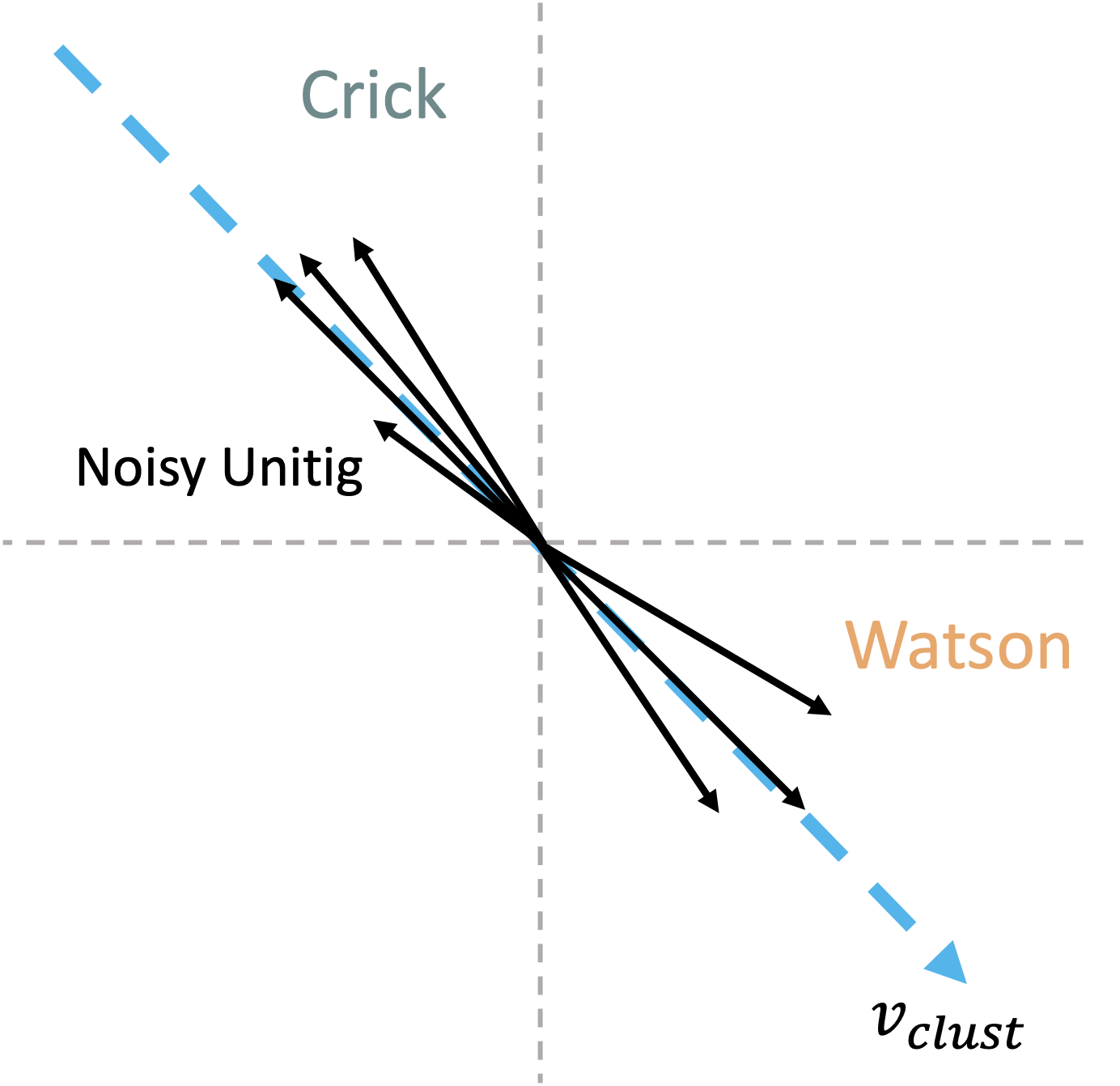


*Fig. S4. Behavior of Absolute SSF Calculated with bwa mem Alignments. Each axis corresponds to a different Strand-seq library. Unitigs from the same chromosome, but in opposite orientation will point in opposite directions along the same cluster vector (blue dashed vector), but are nonetheless clustered together when the absolute cosine similarity is used. A unitig that contains noisy, degenerate sequence is skewed and shrunk towards the 0 vector, but still points along its corresponding cluster vector.*


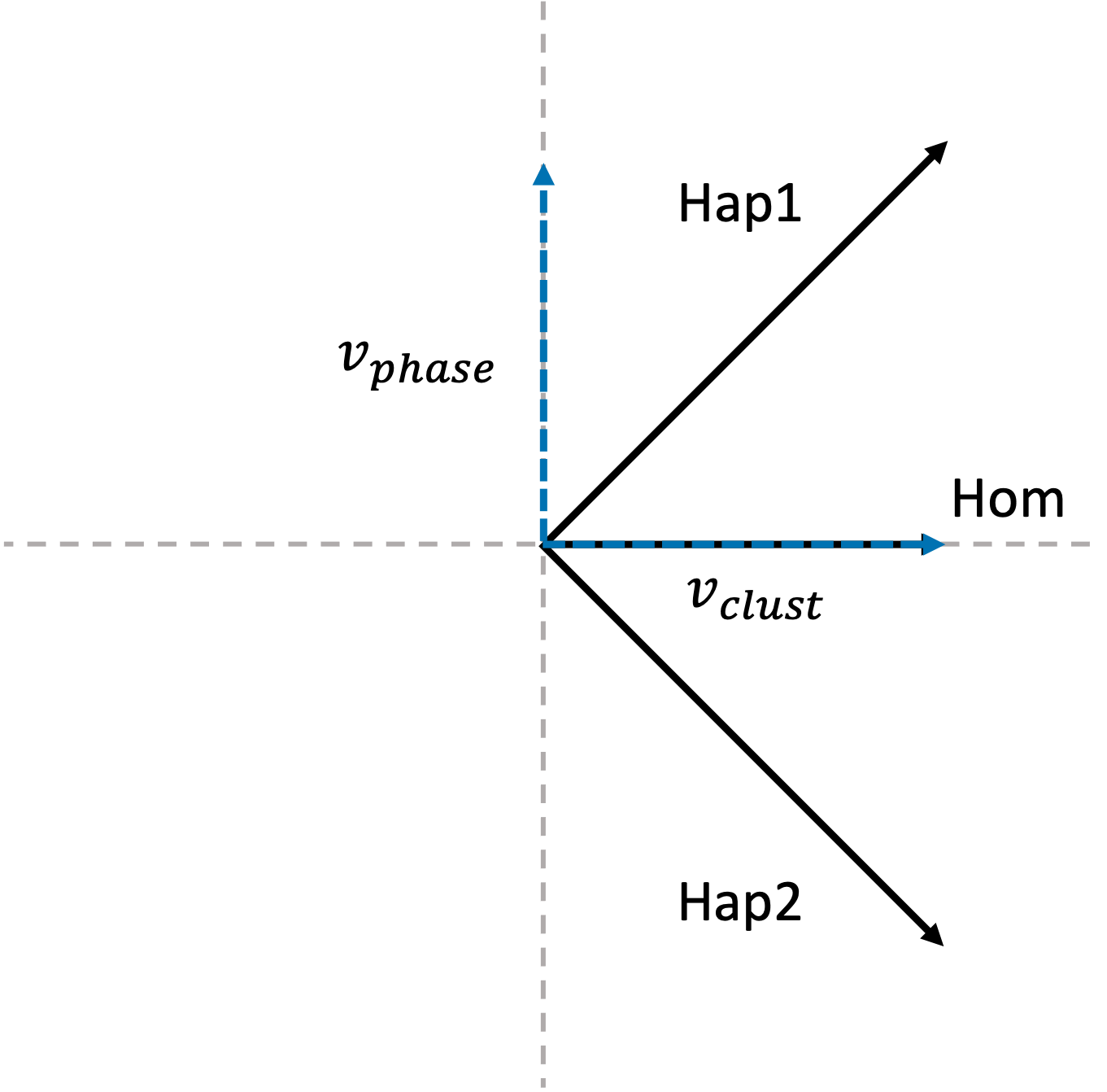


*Fig. S5. Haplotype, Cluster, and Phase Vectors. Each axis corresponds to a different Strand-seq library. When the SSF is calculated with phase-informative reads, the unitig vectors (black arrows) separate from the cluster vector according to haplotype. The difference between the haplotype 1 and haplotype 2 vectors defines the phase vector, which is used to pool Strand-seq libraries. The vectors of unitigs that contain homozygous sequence remain parallel to the cluster vector regardless of the reads used to calculate the SSF.*


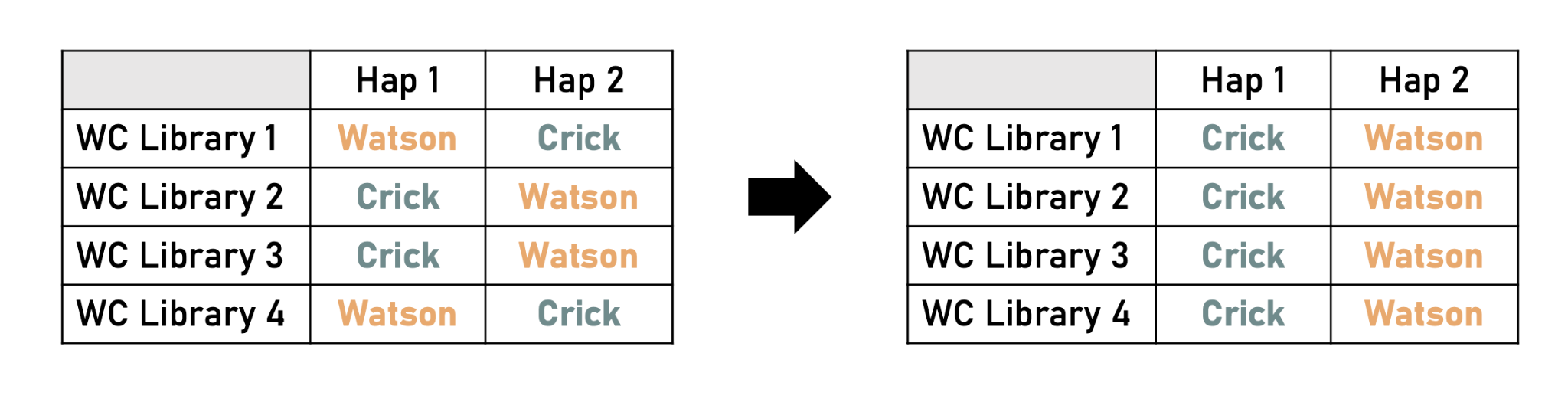


*Fig. S6. Read Labeling for Proper Strand-seq Library Pooling. In each Strand-seq library, the labels (Watson, Crick) of the reads aligning to each haplotype depend on the orientation of the unitigs and the inherited Strand-state of the unmatched strand state library (WC vs. CW library). However, swapping the labels of reads does not affect the steps before library pooling, and so the labeling within each library can safely be treated as if it were arbitrary. Left: Each haplotype receives only Watson or only Crick alignments within each library. However, the pooled library assigns each haplotype a 50/50 ratio of Watson and Crick alignments and fails to reflect the behavior at the individual library level. Right: The labels of libraries 1 and 4 are swapped, and the pooled library now also assigns reads of only one label to each haplotype.*
