# Supplementary material for: Graphasing: phasing diploid genome assembly graphs with single-cell strand sequencing
Source: Genome Biol. 2024 Oct 10;25:265. doi: 10.1186/s13059-024-03409-1 (PMC11466045; doi:10.1186/s13059-024-03409-1)
Supplement: Supplementary file 3 — Supplementary Material 3. Review history [file 13059_2024_3409_MOESM3_ESM.docx]

**1^st^ round**

**Reviewer 1**

Henglin et al. introduce "Graphasing", a workflow that phases genome assembly graphs obtained from a combination of long read sequencing data types into chromosome-scale haplotypes by making innovative use of Strand-seq data. At the core of the workflow is a somewhat convoluted, unsupervised cosine similarity-based clustering approach that clusters unitigs of the assembly graph by chromosome using Strand-Seq library strand states. Strand-seq read alignments to unitigs are then used to produce a shading of the assembly graph from which haplotypes are created with existing tools. The authors convincingly show their method to perform on par with or better than approaches that use another additional type of data. In particular, Graphasing introduces fewer phasing errors than approaches using Hi-C data and seems as accurate as trio phasing that requires the availablility of genomic samples from both parents of the studied subject. In practice, acquiring Strand-Seq data and phasing of assembly graphs with Graphasing should therefore be considered a viable alternative to Hi-C based assembly graph phasing.

-- Major issues:

1. While the evaluation of Graphasing is overall very convincing, the main results section "Phasing Method Comparison" including tables and figures is quite exhausting to read due to the multi-dimensional evaluation setup: In addition to the three approaches (based on Strand-Seq, Hi-C or trio data), the authors compare two data sets (HG00733 and NA24385), two assembly tools (Verkko and hifiasm), and in some cases the two haplotypes (maternal and paternal) in all 12 (or 24) combinations to each other. Given this setup, parts of the text seem to describe more the differences between assembly tools or data sets rather than highlighting the advantages of Strand-Seq and Graphasing compared to phasing with Hi-C or trio data. Improving the comprehensibility of this key part of the manuscript is necessary in my opinion, e.g. by revising the text and redesigning (e.g. splitting) some tables and figures or by focussing on the most relevant subset of combinations in the main text. As the authors conclude that "Verkko + Graphasing produced the highest-quality haplotypes", I could even imagine to move hifiasm to the supplementary material although less drastic improvements should be considered first.

2. The choice of the cosine similarity for clustering is central to the Graphasing workflow and represents a methodologic contribution beyond chaining existing tools into a workflow. It is very well justified and makes perfect sense. I am wondering, why the initial clustering is obtained using a "discrete clustering algorithm [64]" from some PhD thesis that occasionally introduces large phasing errors due to its stochasticity. In addition, these initial clusters require several subsequent refinement steps (e.g. small cluster removal and cluster merging) before the final cosine similarity agglomarative clustering. These many steps make the clustering approach difficult to grasp. It would seem more straight-forward and cleaner to me to use cosine similarity-based agglomerative clustering from the start.

3. The stochasticity problem of the initial clustering is mentioned in the methods but not further evaluated in the results section. How frequent and how serious are the "large errors" that were "occasionally" observed. Is there a way to control for this in general use cases? Without a way to validate results, I understand that a single application of the workflow cannot be relied upon to produce correct results.

4. Some parts of the methods seem to be tailored very specifically not just to diploid, but to the human genome. In particular, some of the thresholds and steps appear closely fitted to the data or may have been implemented to solve issues observed in the data at hand. Was the algorithm developed using the same data as used for evaluation? How is it expected to perform on more divergent data, e.g. from mouse genomes?

5. The use of the terms homozygous and heterozygous to refer to strand states is not ideal, as zygosity refers to similarity of genetic sequence. The strand state (WW/CC/WC/CW) differs from that. Unless this is already common Strand-Seq terminology that I have missed so far, I would perfer to avoid overloading the terms, especially in the context of haplotype phasing where they are abundantly used in their original meaning, too.

6. Even though I am an enthusiast of the Strand-Seq technology and its potential for genome analysis, I have to mention here that Strand-Seq data is currently rare and difficult to aquire. This means that Graphasing currently targets a very narrow audience. While previous high-impact Strand-Seq publications presented the technology and applications that answered biomedical questions, this work "only" introduces a computational approach for analysing Strand-Seq data. Its impact will depend on whether Strand-Seq data will become easier to aquire and more abundant in the future or not. I would welcome a hint about Graphasing's target audience in the discussion.


-- Minor issues:

1. In several places (unitig clustering, haploid identification, cosine similarity) where the authors write about the *hetero*zygous strand state is it referred to as *homo*zygouse strand state. This makes the methods hard to follow and calls for another proof-reading of the methods section.

2. Is it guaranteed that the use of the full 192 samples leads to better phasing than 96 high-quality samples or could it be that the 96 low-quality samples introduce noise? In that case, the diminished agreement of the 100% high-quality data set as opposed to the 75% data sets would be wanted. Is it possible to investigate the disagreement on the mentioned 5Mbp unitig further?

3. I do not understand what is meand by the sentence "Only the Verkko NA24385 haplotypes had no gaps detected on unitigs aligning to autosomal chromosomes" on page 13. Is this data that is not shown?

4. Absolute Cosine Similarity Clustering, page 22: Why do you need to account for possible misorientations by using absolute cosine similarity values when using absolute SSF vectors already? Aren't absolute SSF vectors always in the first quadrant such that it is impossible to obtain vectors pointing in opposite direction?

5. I am confused by the use of "haplotype markers". Is this a term that needs a definition or do you simply mean to refer to labelling (marking) one haplotype as 0 and the other as 1?

6. Please indicate chapter and section for reference 64, where to find the discrete clustering algorithm.

7. The section "Runtime and memory usage evaluation" on page 17 should refer to table 3 rather than table 2. The third column of this table should be omitted.

6. How many unitigs remain in the evaluated assembly graphs after the length filtering step?

**Reviewer 2**

In the manuscript, Henglin et al present "Phasing Diploid Genome Assembly Graphs with Single-Cell Strand Sequencing". The key contribution of the manuscript is to introduce "Graphasing", a workflow and software package to leverage the phase signal of Strand-seq to produce chromosome-scale de novo haplotypes for diploid genomes. The authors perform extensive benchmarking in two widely studied samples (NA24385 and HG00733) and find Graphasing performs comparably to gold-standard trio-phasing in contiguity, phasing accuracy, and assembly quality, and outperforms HiC in phasing accuracy. Overall the manuscript is well written and the results are compelling for this important topic.

My main comments center on the nature of the StrandSeq data. The authors present summary results from a Strand-seq Library titration experiment (Figure 8), but this leaves many questions, especially why the 75% datasets produce better assemblies than the 100% dataset. Relatedly how are high-quality/low-quality libraries identified and are there any techniques to filter or correct low quality libraries into high quality libraries. Finally, why are there any errors remaining after using these data - are there regions that are systematically inaccessible, are there errors in the strand-seq data, errors in the analysis, or something else? Would it be possible to combine Strand-seq with HiC or trios (or both) to produce "perfect" assemblies directly from the assembler?

Relatedly, the manuscript focuses on extremely high quality datasets and extremely high quality preliminary assemblies. Similar to the strand-seq downsampling experiments, it would be informative to assess performance with lower quality input assemblies as these are likely to occur in other species that are not as well resourced as human genomes. For example, unitigs shorter than 250kbp seem are now systematically excluded from the analysis, but this will be very problematic for some species/assemblies.

Finally, the manuscript would be improved by briefly explaining the Strand-seq protocol and some of the challenges involved for data production as not all readers will be familiar. The manuscript does include the key references, but it would be preferred to make the manuscript self contained. The manuscript should highlight in the introduction that it can be challenging to produce Strand-seq data. This is currently mentioned in the discussion, but this should be introduced earlier, especially as a counterpoint to the challenges highlighted for collecting trios and/or HiC.

Other comments:

Figure 1 would be improved with text labels for the different panels to quickly state what is represented in each algorithmic phase

**Authors’ response**

Reviewer #1

Henglin et al. introduce "Graphasing", a workflow that phases genome assembly graphs obtained from a combination of long read sequencing data types into chromosome-scale haplotypes by making innovative use of Strand-seq data. At the core of the workflow is a somewhat convoluted, unsupervised cosine similarity-based clustering approach that clusters unitigs of the assembly graph by chromosome using Strand-Seq library strand states. Strand-seq read alignments to unitigs are then used to produce a shading of the assembly graph from which haplotypes are created with existing tools. The authors convincingly show their method to perform on par with or better than approaches that use another additional type of data. In particular, Graphasing introduces fewer phasing errors than approaches using Hi-C data and seems as accurate as trio phasing that requires the availablility of genomic samples from both parents of the studied subject. In practice, acquiring Strand-Seq data and phasing of assembly graphs with Graphasing should therefore be considered a viable alternative to Hi-C based assembly graph phasing.

**Response:** We thanks the reviewer for this positive assessment.

-- Major issues:

1. While the evaluation of Graphasing is overall very convincing, the main results section "Phasing Method Comparison" including tables and figures is quite exhausting to read due to the multi-dimensional evaluation setup: In addition to the three approaches (based on Strand-Seq, Hi-C or trio data), the authors compare two data sets (HG00733 and NA24385), two assembly tools (Verkko and hifiasm), and in some cases the two haplotypes (maternal and paternal) in all 12 (or 24) combinations to each other. Given this setup, parts of the text seem to describe more the differences between assembly tools or data sets rather than highlighting the advantages of Strand-Seq and Graphasing compared to phasing with Hi-C or trio data. Improving the comprehensibility of this key part of the manuscript is necessary in my opinion, e.g. by revising the text and redesigning (e.g. splitting) some tables and figures or by focussing on the most relevant subset of combinations in the main text. As

the authors conclude that "Verkko + Graphasing produced the highest-quality haplotypes", I could even imagine to move hifiasm to the supplementary material although less drastic improvements should be considered first.

**Response:** We thank the reviewer for their insight. We have simplified the presentation of results by presenting each assembly without separating by haplotype, halving the number of data points presented without removing substantive information. Additionally, the text in each results section has been refocused to highlight the differences between each phasing method.

2. The choice of the cosine similarity for clustering is central to the Graphasing workflow and represents a methodologic contribution beyond chaining existing tools into a workflow. It is very

well justified and makes perfect sense. I am wondering, why the initial clustering is obtained using a "discrete clustering algorithm [64]" from some PhD thesis that occasionally introduces large phasing errors due to its stochasticity. In addition, these initial clusters require several subsequent refinement steps (e.g. small cluster removal and cluster merging) before the final cosine similarity agglomarative clustering. These many steps make the clustering approach difficult to grasp. It would seem more straight-forward and cleaner to me to use cosine similarity-based agglomerative clustering from the start.

**Response:** We thank the reviewer for their kind description of our contribution. We concur with their assessment, and in the updated Graphasing algorithm, the stochastic clustering step has been removed, and the cosine-similarity based agglomerative clustering is used from the start. For the assemblies evaluated, this change had minimal effect on the outcome of the clustering, but we anticipate that this will greatly reduce the variance in clustering outcome for less contiguous assemblies.

3. The stochasticity problem of the initial clustering is mentioned in the methods but not further evaluated in the results section. How frequent and how serious are the "large errors" that were "occasionally" observed. Is there a way to control for this in general use cases? Without a way to validate results, I understand that a single application of the workflow cannot be relied upon to produce correct results.

**Response:** We share the reviewer’s methodological concerns. In the updated Graphasing algorithm, the stochastic clustering step has been removed, and the clustering is now entirely deterministic. Accordingly, these sorts of errors are no longer possible.

4. Some parts of the methods seem to be tailored very specifically not just to diploid, but to the human genome. In particular, some of the thresholds and steps appear closely fitted to the data or may have been implemented to solve issues observed in the data at hand. Was the algorithm developed using the same data as used for evaluation? How is it expected to perform on more divergent data, e.g. from mouse genomes?

**Response:** We thank the reviewer for raising this point. The difficulty in evaluating our Graphasing pipeline in other (non-human) organisms is caused by the need for various high-quality sequencing datasets apart from a current Strand-seq dataset. We would like to emphasize that the Strand-seq technology on its own has been successfully employed to study, e.g. mouse (Falconer et al., Nat Meth 2012) or non-human primates (Porubsky et al., Nat Gen 2020) genomes. Our use case nevertheless requires high-quality - and fairly expensive - long-read data ideally from both current state-of-the-art methods (PacBio Hifi and ONT ultralong). This combination of sequencing data is commonly only produced for human samples. Although we agree with the reviewer that we cannot systematically rule out any over-fitting of thresholds and parameters to human data, we do not see a feasible way at this point to evaluate this due to the scarcity of suitable datasets. As these data types continue to drop in price, we anticipate that many such data sets will be produced in the next years.

5. The use of the terms homozygous and heterozygous to refer to strand states is not ideal, as zygosity refers to similarity of genetic sequence. The strand state (WW/CC/WC/CW) differs from that. Unless this is already common Strand-Seq terminology that I have missed so far, I would perfer to avoid overloading the terms, especially in the context of haplotype phasing where they are abundantly used in their original meaning, too.

**Response:** We thank the reviewer for their suggestion. ”Heterozygous” and “homozygous” strand states have been replaced in the manuscript with “unmatched”, corresponding to WC and CW strand states, and “matched”, corresponding to WW and CC strand states.

6. Even though I am an enthusiast of the Strand-Seq technology and its potential for genome analysis, I have to mention here that Strand-Seq data is currently rare and difficult to aquire. This means that Graphasing currently targets a very narrow audience. While previous high-impact Strand-Seq publications presented the technology and applications that answered biomedical questions, this work "only" introduces a computational approach for analysing Strand-Seq data. Its impact will depend on whether Strand-Seq data will become easier to aquire and more abundant in the future or not. I would welcome a hint about Graphasing's target audience in the discussion.

**Response:** Indeed Strand-seq is presently not commercially available and produced only by a limited number of academic labs. At the same time, fairly large bodies of data are available through efforts such as the Human Genome Structural Variation Consortium, in the frame of which we have used Strand-seq in the past (Ebert et al., Science, 2021) and have continued to produce Strand-seq data for human genomes (with 65 genomes being completed at this point, with a corresponding publication in preparation). In our view, the availability of the Graphasing pipeline will increase the appeal of Strand-seq and might promote its adoption. We have added a note to the discussion to point out that we *“consider the Graphasing pipeline to be an attractive assembly method especially for the production of reference-quality genomes when trio data is unavailable.”*

-- Minor issues:

1. In several places (unitig clustering, haploid identification, cosine similarity) where the authors write about the *hetero*zygous strand state is it referred to as *homo*zygouse strand state. This makes the methods hard to follow and calls for another proof-reading of the methods section.

**Response:** We appreciate the reviewer’s feedback and have ensured clarity of the methods in the updated manuscript.

2. Is it guaranteed that the use of the full 192 samples leads to better phasing than 96 high-quality samples or could it be that the 96 low-quality samples introduce noise? In that case, the diminished agreement of the 100% high-quality data set as opposed to the 75% data sets

would be wanted. Is it possible to investigate the disagreement on the mentioned 5Mbp unitig further?

**Response:** We thank the reviewer for their feedback regarding the lack of clarity in the evaluation of the titration experiment. In the updated manuscript, the titration library sets are instead evaluated through alignment against the gold-standard HG002 v1.0 reference, allowing performance to be more directly evaluated. With this new evaluation, performance continues to improve with increasing percentage of high-quality libraries in the library set.

3. I do not understand what is meand by the sentence "Only the Verkko NA24385 haplotypes had no gaps detected on unitigs aligning to autosomal chromosomes" on page 13. Is this data that is not shown?

**Response:** We appreciate the reviewer pointing out this confusing sentence. In the current description of the results, which was rewritten to account for the updated Graphasing, the sentence has been deleted.

4. Absolute Cosine Similarity Clustering, page 22: Why do you need to account for possible misorientations by using absolute cosine similarity values when using absolute SSF vectors already? Aren't absolute SSF vectors always in the first quadrant such that it is impossible to obtain vectors pointing in opposite direction?

**Response:** The reviewer was right in their confusion, as this point was falsely expressed, and we thank them for catching this. This sentence has been removed, and the description of clustering has been clarified in the updated manuscript. Additionally, Figure 1, panel B, and the corresponding supplemental figures have been updated to more clearly illustrate the clustering process.

5. I am confused by the use of "haplotype markers". Is this a term that needs a definition or do you simply mean to refer to labelling (marking) one haplotype as 0 and the other as 1?

**Response:** We have added a couple sentences describing haplotype markers at the end of the section “Haplotype Informative Strand-seq Library Pooling”. The sentences are reproduced below as well:

*“The pooled reads constitute a pair of haplotype marker counts for each unitig, whose values indicate the haplotype to which the unitig belongs. Unitigs specific to a given haplotype will have a high marker count for the corresponding haplotype, and a low marker count for the opposite haplotype, while homozygous unitigs are expected to be assigned a balanced number of markers for both haplotypes.”*

6. Please indicate chapter and section for reference 64, where to find the discrete clustering algorithm.

**Response:** In the updated Graphasing algorithm, the stochastic clustering step has been removed.

7. The section "Runtime and memory usage evaluation" on page 17 should refer to table 3 rather than table 2. The third column of this table should be omitted.

**Response:** We thank the reviewer for catching this error + suggestion and have updated the manuscript accordingly.

8. How many unitigs remain in the evaluated assembly graphs after the length filtering step?

**Response:** We have added a new supplementary table (S1) containing this information. The table is reproduced below as well:

| **Sample** | **Assembler** | **Total Unitigs**  **Unitigs < 50kbp**  **Total Assembly**  **(n)**  **(n)**  **Size < 50kbp**  **(%)** |
| --- | --- | --- |
| NA24385 | Verkko hybrid | 8,627 4,966 2.2 |
| NA24385 | hifiasm hybrid | 12,520 4,355 2.5 |
| HG00733 | Verkko hybrid | 1,882 1,353 0.5 |
| HG00733 | hifiasm hybrid | 4,579 2,026 1.2 |

Reviewer #2

In the manuscript, Henglin et al present "Phasing Diploid Genome Assembly Graphs with Single-Cell Strand Sequencing". The key contribution of the manuscript is to introduce "Graphasing", a workflow and software package to leverage the phase signal of Strand-seq to produce chromosome-scale de novo haplotypes for diploid genomes. The authors perform extensive benchmarking in two widely studied samples (NA24385 and HG00733) and find Graphasing performs comparably to gold-standard trio-phasing in contiguity, phasing accuracy, and assembly quality, and outperforms HiC in phasing accuracy. Overall the manuscript is well written and the results are compelling for this important topic.

**Response:** We thank the reviewer for this positive assessment of our work.

My main comments center on the nature of the StrandSeq data. The authors present summary results from a Strand-seq Library titration experiment (Figure 8), but this leaves many questions, especially why the 75% datasets produce better assemblies than the 100% dataset.

**Response:** We thank the reviewer for their feedback regarding the lack of clarity in the evaluation of the titration experiment. In the updated manuscript, the titration library sets are instead evaluated through alignment against the gold-standard HG002 v1.0 reference, allowing performance to be more directly evaluated. With this new evaluation, performance continues to improve with increasing percentage of high-quality libraries in the library set.

Relatedly how are high-quality/low-quality libraries identified and are there any techniques to filter or correct low quality libraries into high quality libraries.

**Response:** We thank the reviewer for highlighting this point. Originally, the curation and manual selection of high-quality Strand-seq libraries required a domain expert to visually evaluate certain characteristics of each library such as sufficient read depth and adequate strand-specific signal, i.e. a well-behaved distribution of reads aligning in forward or reverse direction (the Crick/Watson signal in Strand-seq jargon) to the reference genome. This laborious process is usually performed twice to reduce the effect of the “human component” to obtain more robust quality labels for the entire dataset. This manual selection process represented a major bottleneck, which is why we decided to automate the library selection employing robust machine learning technology (Gros et al., ASHLEYS: automated quality control for single-cell Strand-seq data. *Bioinformatics* 2021). We have added a pointer to this paper describing the process in detail as a technical reference to our manuscript.

Finally, why are there any errors remaining after using these data - are there regions that are systematically inaccessible, are there errors in the strand-seq data, errors in the analysis, or something else?

**Response:** We thank the reviewer for raising this relevant question. On the one hand, no sequencing technology is perfect and there is always a certain amount of inherent technological

noise in the data. On the other hand, there are algorithmic limitations to aligning short reads, i.e. finding the presumably "correct" and unambiguous placement for each Strand-seq read is not trivial - if at all possible - in difficult sequence contexts such as highly repetitive regions that, unfortunately, make up a lot of sequence in the human genome. So in order to successful phase a genomic region, the long reads used to create the assembly graph have to create sufficiently long node sequences to cover regions where short reads can be mapped unambiguously. We now mention the importance of being able to map short Strand-seq reads in the discussion to emphasize the point that the reviewer raised.

Would it be possible to combine Strand-seq with HiC or trios (or both) to produce "perfect" assemblies directly from the assembler?

**Response:** In principle, that would be possible, but all these technologies share the limitation of being based on short reads which are either aligned (Hi-C, Strand-seq) or used through the contained k-mers (trio phasing). So our anticipation would be that such an ensemble approach might resolve some problems, but the at the same time we would caution that for a “perfect” assembly the input assembly graph needs to be “perfect”, which can only be achieved by improving the input long read data times (and potentially the software tool producing the assembly graph).

Relatedly, the manuscript focuses on extremely high quality datasets and extremely high quality preliminary assemblies. Similar to the strand-seq downsampling experiments, it would be informative to assess performance with lower quality input assemblies as these are likely to occur in other species that are not as well resourced as human genomes. For example, unitigs shorter than 250kbp seem are now systematically excluded from the analysis, but this will be very problematic for some species/assemblies.

**Response:** We thank the reviewer for their question. To evaluate the performance when only one long read data type is available, we constructed an assembly graph using only Hifi data (44.5x coverage) for NA24385 with hifiasm and phased it using Graphasing and trio. Overall, the assemblies are still of very good quality, with N50s >57Mbp, QVs >55 and switch error rates <0.23% for both Graphasing and trio. The detailed evaluation results are shared in Supplemental Table S4 and reproduced below. For this more fragmented assembly, Graphasing produced haplotypes with slightly worse contiguity and phasing accuracy, in contrast to the hybrid assemblies where Graphasing and trio performed nearly identically on these metrics. At the same time, Graphasing produced haplotypes with slightly higher QV, and somewhat more events reported by paftools misjoin, a pattern matching the results of the hybrid assemblies. For both these and the hybrid assemblies, the Graphasing haplotypes also had a higher rate of gene missingness than the trio assemblies, but as mentioned in the corresponding results section, the evaluation of HaPU assemblies is inflated relative to trio for these metrics. Our overall assessment, which we stated at the end of the section “Phasing Method Comparison” was that: “*Graphasing can also produce high-quality haplotypes from hifiasm hifi-only (44.5x) assemblies, which are competitive with trio phased-assemblies for NA24385.”*

| **Evaluation** | **Metric Graphasing** | **Trio** |
| --- | --- | --- |
| Contiguity | auN (Mbp) 66.0 | 67.9 |
| Contiguity | N50 (Mbp) 57.8 | 64.9 |
| yak trioeval | Switch Error Rate (%) 0.23 | 0.16 |
| yak trioeval | Hamming Error Rate (%) 0.57 | 0.15 |
| yak qv | QV 56.1 | 55.8 |
| paftools misjoin | Gaps [acrocentric-only] (n) 7 [0] | 2 [0] |

paftools misjoin Interchromsomal Misjoins [acrocentric-only] (n) 0 [3] 0 [4] paftools misjoin Inversions [acrocentric-only] (n) 1 [0] 0 [0] paftools asmgene MMC (%) 12.0 1.4 paftools asmgene MSC (%) 1.4 0.2

Finally, the manuscript would be improved by briefly explaining the Strand-seq protocol and some of the challenges involved for data production as not all readers will be familiar. The manuscript does include the key references, but it would be preferred to make the manuscript self contained. The manuscript should highlight in the introduction that it can be challenging to produce Strand-seq data. This is currently mentioned in the discussion, but this should be introduced earlier, especially as a counterpoint to the challenges highlighted for collecting trios and/or HiC.

**Response:** We thank the reviewer for their suggestion. The introduction has been restructured and a description of the Strand-seq protocol as well as comparisons to trio and Hi-C have been added at the end of the “Background” section.

Other comments:

Figure 1 would be improved with text labels for the different panels to quickly state what is represented in each algorithmic phase

**Response:** We thank the reviewer for their suggestion and have updated Figure 1 with labels for each facet.

**2^nd^ round**

**Reviewer 2**

The authors have addressed my previous concerns. I especially appreciate the algorithmic improvements to make the analysis more robust and deterministic and demonstrating the performance on lower quality input assemblies
